# The Role of Worked Examples in Supporting Early Math Learning

**DOI:** 10.3390/jintelligence14090192

**Published:** 2026-09-01

**Authors:** Taylor Stone, Lesenia R. Fish, Nadia Chernyak, Gabriella Guadalupe, Sara Cordes

**Affiliations:** 1 Department of Psychology and Neuroscience, Boston College, Chestnut Hill, MA 02467, USA; cordess@bc.edu; 2 Department of Psychology, Emory University, Atlanta, GA 30322, USA; lesenia.fish@emory.edu; 3 Department of Cognitive Sciences, University of California, Irvine, CA 92697, USA; nchernya@uci.edu; 4 Department of Newborn Medicine, Boston Children’s Hospital, Boston, MA 02115, USA; gabriellaguadalupe17@gmail.com

**Keywords:** worked examples, division, early childhood, numerical cognition, preschool, mathematical learning

## Abstract

Worked examples—step-by-step examples of how to solve a problem correctly or incorrectly—are known to promote algebraic thinking in middle and high school students. Whether they are useful in facilitating math learning in much younger students is unclear, however. The present study investigated how highlighting correct versus incorrect division strategies in a sharing context supports preschool children’s learning and transfer across division tasks. Two hundred and one children (ages 4.01–6.96 years, *M* = 5.57) viewed videos of agents dividing toys using either a correct (one-to-one turn-taking) or incorrect (i.e., error prone) strategy. The children were asked to explain what the agent had done correctly, what the agent had done incorrectly, or to simply identify the color of the agents’ shirts (Baseline condition). Afterwards, they completed two division tasks which required them to divide resources between two recipients (similar to training) and then between three recipients (novel division task). The results revealed that highlighting the correct or incorrect procedures led to improved performance on the division task relative to baseline, particularly for the youngest children completing the most challenging trials. Moreover, analyses of children’s explanations revealed that those children who spontaneously referenced the division *process* outperformed those who referenced the outcome or another aspect. These findings suggest that drawing attention to procedural strategies can facilitate division learning in early childhood, highlighting the importance of process-focused instruction as a mechanism for supporting early mathematical development.

## 1. Introduction

Critical to children’s early education is the development of basic mathematical skills. Early numerical understanding is a key component of school readiness, such that children with stronger early math skills achieve greater long-term success in academics. For example, children’s numerical competencies in kindergarten and first grade strongly predict their mathematics achievement through third grade (Jordan et al., 2009). Moreover, math skills assessed prior to formal schooling have been found to be the single strongest predictor of later academic performance, even relative to early reading and attention skills (Duncan et al., 2007). Encouraging children to engage with numerical content through guided instruction or play lays essential groundwork for building numerical competence in early education environments (Aubrey et al., 2006; DePascale & Ramani, 2025).

Given the importance of early math skills, it is critical to identify ways to promote mathematical learning in young children. One proven strategy that has been used with middle and high school students learning algebra is presenting worked examples, where students are provided step-by-step examples of how to solve a problem either correctly or incorrectly (Renkl, 2014). These worked examples have been shown to promote learning above and beyond that of straightforward instruction (Sweller & Cooper, 1985). By providing students with a worked-out solution, they can focus on understanding the underlying procedure rather than expending their cognitive resources to come up with a solution on their own (Sweller, 2011). In the current study, we explored whether worked examples can be an effective strategy for promoting math learning of division concepts in much younger, preschool-aged children. We introduced children to whole-number division in a socially meaningful context of sharing and highlighted either correct or incorrect sharing strategies. In doing so, we tested whether directing attention to distinct procedural strategies can support children’s understanding of division and fairness during early childhood.

### 1.1. Sharing and Division

Division is an important foundational math skill, important for success with proportional reasoning, fractions, and algebraic thinking (DeWolf et al., 2015). Although formal instruction of division is typically introduced in third grade in the United States (Common Core State Standards Initiative, 2010), research has suggested that children as young as 5 years old can reason about and engage in numerical division even prior to formal schooling (e.g., Gilmore & Spelke, 2008; McCrink & Spelke, 2016). Despite its importance for later math success, both children and adults tend to show a relatively weak conceptual understanding of division, highlighting the need to find ways to support early division learning (Dubé & Robinson, 2018). One promising way to support this concept is to present division problems in a meaningful, real-world context. Recent work has shown that children’s understanding of division is stronger when it has been embedded in social contexts, such as sharing resources, with this benefit specific to division rather than arithmetic more broadly (Hamamouche et al., 2020).

Sharing contexts are among the earliest situations in which children encounter division-related problems. Sharing and division tasks have many similarities in their structure such that they both require that individuals attend to the total number of items (i.e., the set size) and the number of recipients (Squire & Bryant, 2002a, 2002b). To share items equally, children must be able to track quantities across multiple recipients, compare set sizes, and distribute items to produce equal outcomes (Squire & Bryant, 2002b). Although even infants show expectations for equality in third-party distributions (Geraci & Surian, 2011; Sommerville et al., 2013), younger children often struggle to produce equal distributions when sharing on their own. It is not until children master the verbal counting procedure that they can reliably generate equal outcomes in sharing contexts (Chernyak et al., 2016, 2020; Sohail et al., 2022), despite preschool-aged children demonstrating a conceptual understanding of what it means to share fairly (Smith et al., 2013; Sarnecka & Wright, 2013). Importantly, as children’s numerical knowledge develops, they increasingly recognize counting as an intentional and effective strategy for ensuring fair outcomes (Jacobs et al., 2022).

Together, this work suggests that sharing contexts provide a valuable window into how children apply emerging numerical knowledge to division-related problems (Hamamouche et al., 2020). Because sharing requires attention to both the outcome and the procedure, it serves as a particularly useful context for examining how children evaluate and learn different division strategies. Thus, in the current study, we chose to use the context of sharing to explore whether highlighting correct and incorrect division procedures—through worked examples—can improve preschool children’s understanding of division.

### 1.2. Worked Examples and Cognitive Load in School-Aged Mathematics Learning

Most research on worked examples has focused on older, middle- and high-school-aged learners. Mistakes in mathematics are quite common, and rather than avoiding mistakes, research has highlighted that mistakes can be used as a teaching opportunity when they are accompanied by appropriate instructional support (Barbieri et al., 2021). One strategy that has been effective for math learning is the use of worked examples: presenting fully solved, step-by-step examples of similar math problems to draw attention to specific aspects of the procedure to promote math learning (Renkl, 2014; Ngu et al., 2025). For example, in a multi-digit subtraction problem, an incorrect worked example may show a solved problem in which the solver forgot to carry a one during one of the steps of the procedure. Identifying this error requires the learner to track each step of the procedure to see where the mistake occurred. Early work by Sweller and Cooper (1985) found that children learned more effectively by observing how math problems were solved than when they solved problems independently, particularly when the content was new. Subsequent research has suggested that worked examples are particularly effective for novice learners because they can help reduce cognitive load by breaking down complex math problems into smaller, meaningful steps, allowing students to focus on understanding the underlying principles, rather than answering the question independently (Sweller, 2006; Renkl, 2014; Chen et al., 2023). Moreover, meta-analytical evidence has indicated that worked examples reliably improved math performance compared to just solving practice problems, with these effects being stronger during the early stages of learning (Barbieri et al., 2023). Together, this work suggests that worked examples provide an efficient and effective instructional approach for supporting the development of more advanced math concepts.

Research on worked examples has focused on two different approaches. One approach is to present worked examples involving errors (incorrect examples). This type of example challenges students to identify the mistake and understand why it is incorrect and then determine how the solution can be corrected (Siegler, 2002; Große & Renkl, 2007). Ohlsson’s (1996) theory of learning suggests that explaining why a solution is incorrect can help learners refine their problem-solving strategies. By explicitly drawing attention to these procedural mistakes, incorrect worked examples are thought to strengthen both conceptual and procedural knowledge. However, evidence suggests that the effectiveness of incorrect worked examples may depend on the learners’ prior knowledge. When students lack a sufficient understanding of the relevant procedures, they struggle to recognize and explain the mistakes or they may encode incorrect strategies, which limits the benefit of this approach (Große & Renkl, 2007; Ngu et al., 2025).

The other approach is to use correct worked examples, in which students are presented with the correct step-by-step procedure to solve the problem. In this case, students are not required to identify any errors but are simply able to follow how correct procedures can be used to solve the problem. Evidence suggests that this approach may be especially beneficial for students with lower prior knowledge, as it reduces the need to find a solution on their own and allows them to focus on the problem one step at a time (Renkl, 2014; Sweller, 2006; Ngu et al., 2025). A recent meta-analysis of 55 different studies found that correct worked examples led to the greatest improvements in math performance for school-aged children compared to incorrect examples or mixed approaches (Barbieri et al., 2023). These findings suggest that while incorrect worked examples can be conceptually informative for students, correct worked examples may provide more robust learning benefits, particularly for learners who are still developing important mathematical knowledge.

Cognitive Load Theory (CLT) offers one explanation for how and why worked examples are effective, particularly for learners with limited prior knowledge. CLT is a theory that distinguishes between the limited capacity of working memory and the relatively unlimited capacity of long-term memory (Sweller, 2011; Paas & van Merriënboer, 2020). Learning is understood as the process of constructing and refining schemas in long-term memory, which can then be retrieved to help with problem solving. When learners are first exposed to strategies for solving a new type of problem, their working memory can quickly be overloaded, making it difficult for those strategies to be integrated into their long-term memory (Paas & Ayres, 2014; van Merriënboer & Sweller, 2005). In contrast, worked examples lower this cognitive demand by allowing learners to observe the step-by-step procedures, which can then be incorporated into their existing knowledge base (Sweller & Cooper, 1985; Renkl, 2014; Ngu et al., 2025). As a result, worked examples are particularly effective during early learning when learners have not yet developed the schemas necessary to independently solve these complex problems.

Vygotsky’s theory of the Zone of Proximal Development (ZPD) provides a complementary developmental account for how scaffolding can help support learning. The ZPD is defined as the distance between what a learner can accomplish on their own and what they can accomplish with guidance from a more knowledgeable other (Vygotsky, 1978). Learning within the ZPD occurs when this instructional guidance is appropriately tailored to the learner’s current level of understanding, allowing them to engage with tasks that would otherwise be too cognitively demanding to complete alone. From this perspective, tasks that are too simple offer little opportunity for learning, while tasks that are far beyond a learner’s capabilities may also slow down their learning. This guided instruction within the ZPD allows students to engage with new strategies through this structured support and gradually learn new concepts over time. Prior research has shown that children learn more effectively through guided instruction than from discovering new concepts on their own, highlighting the importance of providing learners with adequate support during these stages of early learning (Kirschner et al., 2006).

Critically, CLT and the ZPD offer an explanation for why correct and incorrect worked examples might function differently depending on a learner’s prior knowledge. A correct worked example reduces cognitive load by modeling an error-free example, whereas an incorrect worked example requires that the learner look at each step and identify the mistake, a more demanding task. Together, these theories offer a unifying framework for understanding when and how worked examples are most effective. CLT emphasizes the importance of reducing unnecessary cognitive load, particularly for early learners, while the ZPD highlights the role of guided scaffolding to allow students to engage with tasks beyond their individual capabilities. Taken together, these theories suggest that worked examples should be most beneficial when they appropriately balance cognitive demands with instructional support, which may be especially critical during early childhood when foundational numerical concepts are still being developed. Given that sharing contexts require children to attend to both outcomes and procedures, they provide a natural bridge to these educational approaches that explicitly highlight procedural information, such as worked examples.

### 1.3. Worked Examples in Early Math Learning

Even before children master counting, they show a sensitivity to numerical procedures and can recognize correct versus incorrect counting sequences (Gelman & Meck, 1983) as well as understand that counting can be used as a strategy to solve simple math problems (Wynn, 1992). More specifically, three- and four-year-old children can recognize correct and incorrect counting when observing a puppet count sets of items ranging from 6 to 20 (Gelman & Meck, 1983). As children’s numerical skills develop, they increasingly use their number knowledge to reason about quantity and equality, all core components of early division. This early sensitivity to procedures suggests that young children may be able to evaluate the strategies of another person sharing before they are able to reliably use those strategies effectively themselves. Thus, worked examples, which require evaluating procedures rather than independently solving problems, may be a developmentally appropriate form of instructional support for early learners. Yet whether these benefits extend to preschool-aged children learning foundational concepts such as division remains unclear, and the present study was designed to address this gap.

### 1.4. The Present Study

In this study, we investigated whether worked examples can have similar impacts in math learning in young children. In particular, we explored whether drawing attention to correct and incorrect division procedures can support children’s abilities to divide items equally between multiple recipients. To address this question, four-to-six-year-old children watched videos of agents distributing resources between two recipients. One agent used a correct division strategy, by distributing items using a one-to-one turn-taking strategy. The other agent invoked an incorrect (i.e., error-prone) division strategy, by skipping turns and distributing multiple items at the same time to each recipient. Importantly, the outcome of the sharing was always equal, regardless of the sharing strategy used. After viewing the videos, the children were either asked to explain (1) the correct sharing strategy, (2) the incorrect sharing strategy, or (3) simply to indicate what color shirt the agents were wearing (baseline). Then, the children were asked to divide a different number of items themselves between two other recipients (similar to the agent in the video), and then between three other recipients (a novel number of recipients). Our goal was to determine whether these explanatory prompts influenced children’s performance on the sharing task, and whether drawing attention to either correct or incorrect sharing strategies improved sharing performance relative to a condition in which they simply witnessed sharing but the procedure was not highlighted.

Based on prior research on worked examples and cognitive load, we predicted that drawing attention to either correct or incorrect sharing procedures would improve children’s performance on subsequent division tasks relative to a baseline condition in which procedures were not discussed (Renkl, 2014; Sweller & Cooper, 1985). However, because identifying and learning from incorrect worked examples requires greater prior knowledge and the ability to recognize mistakes, we expected some age-related differences in the effectiveness of these strategies.

Finally, because worked examples have been found to be most effective when the cognitive load is higher, we included sharing trials that varied in difficulty (Paas & Ayres, 2014; Fyfe & Brown, 2018). We expected that the benefits of worked examples would be most evident in trials that were near the upper limits of children’s current abilities—closest to the child’s Zone of Proximal Development (Vygotsky, 1978)—rather than in the easiest trials or for the oldest children.

In addition to using worked examples, we incorporated self-explanation prompts because prior work suggests that actively explaining a solution can deepen learning from both correct and incorrect examples (Siegler & Chen, 2008; Rittle-Johnson, 2006). Rather than requiring children to solve a math problem independently, self-explanation provides scaffolding that may allow them to evaluate and reflect on the division process before they can reliably solve division problems on their own. Although self-explanation has been shown to enhance learning from worked examples in older, school-aged children, little is known about whether prompting preschool-aged children to explain correct and incorrect solutions can support early division learning. The present study addresses this gap by examining whether preschool-aged children’s attention to the procedural aspects of sharing through these explanations strengthens their understanding of division and fairness.

The research questions and hypotheses are as follows:

**RQ1:** Does highlighting correct or incorrect division procedures through worked examples and explanation prompts improve preschool-aged children’s division performance relative to a baseline condition in which the procedure is not explicitly highlighted?

**H1.**  *We hypothesize that children in the Correct and Incorrect conditions will show improved division performance (lower Equality Deviation scores) relative to children in the Baseline condition.*

**RQ2:** Does the effectiveness of correct versus incorrect worked examples vary with age?

**H2.**  *We expect that younger children will benefit more from correct worked examples, whereas older children will benefit more from incorrect worked examples.*

**RQ3:** Does the benefit of worked examples depend on task difficulty?

**H3.**  *The benefit of worked examples was predicted be most apparent in trials near the upper limit of children’s current abilities, rather than in easier trials or among older children who have already mastered division.*

**RQ4:** Does children’s reference to the division process, rather than the outcome, in the explanations relate to their performance?

**H4.**  *We predict that children who reference the process in their explanations will perform more accurately (lower Equality Deviation scores) than children who reference the outcome or something completely unrelated.*

## 2. Method

### 2.1. Participants

Two hundred and one four-to-six-year-old participants were included in the final dataset (mean age = 5.57 years, age range = 4.01–6.96 years). There were 99 girls, 87 boys, 1 non-binary child, and 14 children whose gender was not disclosed. Participants were recruited from the lab’s database of families formed from local birth records in Massachusetts and online postings on social media who completed the study over Zoom (*n* = 147) and from local schools or children’s museums in the greater Boston area who participated in person (*n* = 54). Online participants were compensated with a $5 gift card, and in-person participants received a small gift.^1^ An additional 33 participants were excluded from the final dataset due to technical/video issues (*n* = 19), the child being too distracted (*n* = 7), experimenter error (*n* = 4), and parental interference (*n* = 3).

Among those who provided demographics information (*n* = 114), 65% identified as White or Caucasian, 15% as Asian, 2% as Black or African American, and the remaining participants identified as other multiracial backgrounds or another racial background. Among the primary caregivers, the highest level of education reported was a doctorate degree (19.3%), master’s degree (43.8%), bachelor’s degree (29.8%), and less than a bachelor’s degree (4.4%) (three parents chose not to respond).

### 2.2. Procedure

This study was administered via a computer program in-person or via Zoom. Parents provided informed consent electronically before their child participated.

#### 2.2.1. Worked Example Manipulation

**Figure 1:**
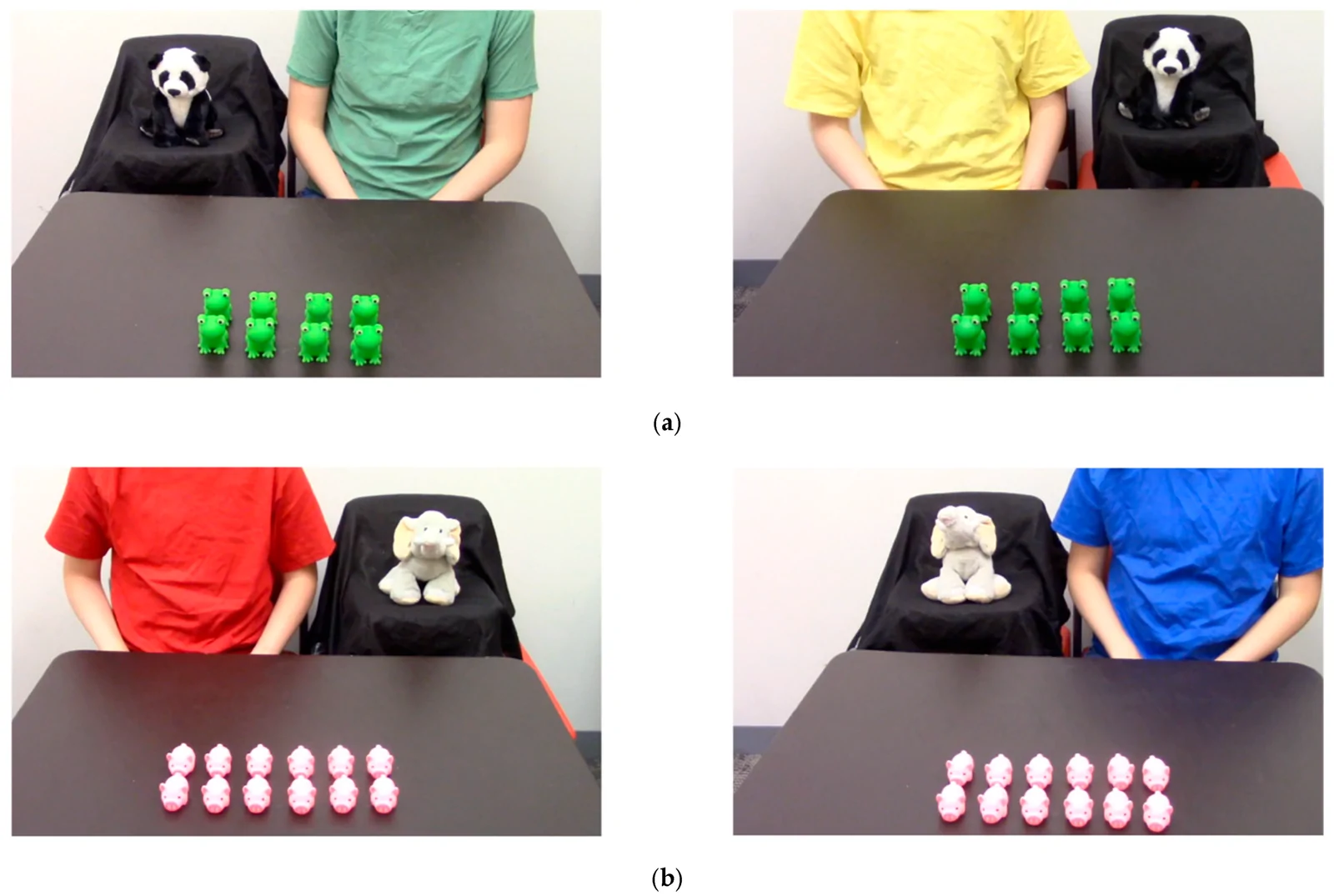
Screenshots from the worked example videos; in one video, participants viewed a correct distribution procedure, and in the other, they viewed an incorrect distribution procedure. The first image (**a**) shows the 8-item trials, and the following image (**b**) shows the 12-item trials.

Participants were first randomly assigned to one of three conditions: Correct (*n* = 68), Incorrect (*n* = 63), or Baseline (*n* = 70). Then, all participants saw two short videos featuring adult agents named Jamie and Alex^2^. Each video showed an agent dividing 8 toy frogs between themselves and “Panda”, a stuffed panda bear seated in a chair next to the agent (see Figure 1). The two videos differed in the *procedure* used to distribute the toys, though this distinction was never labeled for the children; rather, participants were simply told they would watch videos of two people sharing. One agent used a procedurally correct distribution strategy whereas the other used a procedurally incorrect distribution strategy. In both videos, the outcome of the distributions was equal, but the process by which the toys were distributed differed. The order of viewing of the correct and incorrect procedure videos was counterbalanced across participants.

In the procedurally correct video, the agent used a turn-taking strategy to move one toy at a time while counting the items aloud—first placing one toy in front of themselves and counting “one”, then one in front of Panda and counting “one”, then placing a second toy in front of themselves and counting “two”, and so on until all toys were distributed equally between themselves and Panda. In the procedurally incorrect video, the agent appeared to follow this same turn taking strategy and counted aloud as if they were sharing evenly (e.g., “1, 1, 2, 2, 3, 3, 4, 4”, etc.), but during one of the turns, the agent failed to move a toy in front of the intended recipient while still counting the item. On some trials, this skipped action occurred when the agent was supposed to place a toy in front of Panda, and on other trials when the agent was supposed to place a toy in front of themselves; this manipulation was counterbalanced across participants. Later in the process, the agent compensated for this action by moving over two toys at once (again while only counting a single item), resulting in an equal final distribution, despite the procedural error. Whether the agents first placed the toy in front of themselves or Panda was counterbalanced across participants (see OSF for videos: https://osf.io/vsbgh/overview?view_only=54554e96dfac4788adf6d51a8069ed79 (accessed on 6 April 2026)).

After viewing both videos, participants were asked identification questions. First, they were asked, “One of these people made a mistake when they were sharing. Can you point at who made a mistake when they were sharing?” If the participant answered incorrectly, they were corrected, then both videos were replayed in full, and they were asked the question again. Then participants were asked, “And one of these people shared the right way and did NOT make a mistake when they were sharing. Can you point at who shared the right way?”. Again, if the participant answered incorrectly, the participant was corrected, both videos were replayed, and the questions were asked again.

Based on their assigned condition, participants were asked an open-ended follow-up question to probe their understanding or attention to the division process. In the Correct condition, participants were asked to describe what the agent did to share correctly. In the Incorrect condition, participants were asked to describe what the agent did to share incorrectly. In the Baseline condition, participants were asked to identify the color of the shirt each agent was wearing.

Participants then viewed a second block of videos in which the agents shared 12 items. In this block, the agents were named Taylor and Jordan, and they distributed toy pigs between themselves and a different recipient, a stuffed elephant named Ellie. The structure of this block was identical to the first. The order of the correct and incorrect videos was counterbalanced independently of the order in the first block, such that the video order in the second block was not yoked to the order presented in the first block. Again, participants were asked the same identification questions and condition-specific follow-up questions after viewing the videos. The only manipulation that differed by condition was the content of the follow-up questions asked after viewing the videos.

The incorrect worked example video is modified slightly from the classic definition of an “incorrect” worked example in the school-age literature (e.g., Sweller & Cooper, 1985; Renkl, 2014), in which a procedural error typically leads to an incorrect final answer. We instead designed our incorrect example so that the error was corrected by the end of the video, yielding the same equal outcome as the correct example. This is because preschool-aged children are known to weigh outcomes heavily when evaluating fairness and are still developing sensitivity to procedure. Had our incorrect example produced an unequal outcome, children’s responses would likely have reflected outcome-based reasoning rather than a genuine evaluation of the sharing procedure, confounding our central manipulation.

#### 2.2.2. Test Trials

After the worked example manipulation, participants completed a sharing/division task on the computer. This task included six division trials across two blocks: Similar and Dissimilar. The order of the trials within both the Similar and Dissimilar blocks was randomized, but the Similar block was always presented before the Dissimilar block. The Similar block was intentionally presented first because its two-recipient structure directly mirrored the worked example videos the children had just viewed, allowing the children to first apply what they had observed to a familiar format before extending it to the newer, more demanding, three-recipient structure of the Dissimilar block.

**Figure 2:**
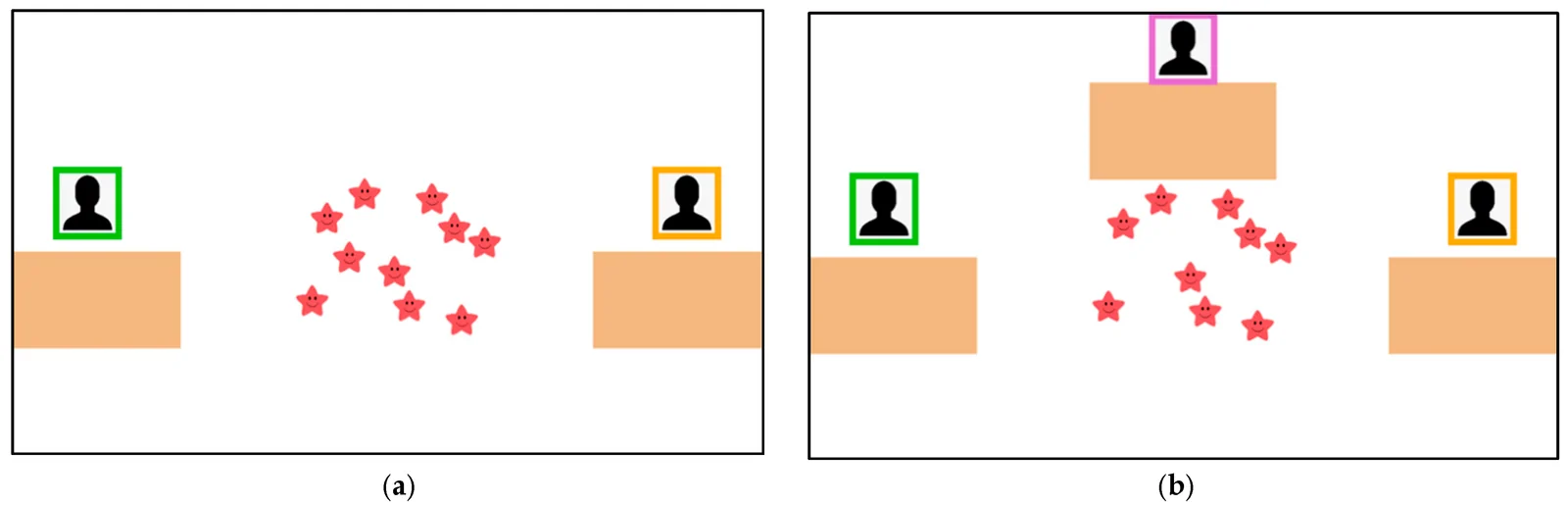
Example of the division task (medium difficulty level) in the (**a**) Similar block and the (**b**) Dissimilar block.

In the Similar block, children were instructed to share stickers with two recipients, a recipient (visually presented as a silhouette of a head, see Figure 2) outlined in green and a recipient outlined in orange, and to share the stickers so that each person has the same amount. There were 3 trials in the Similar block, in which the number of items to be shared varied from 6 (easy), 10 (medium), to 12 (hard) stickers (order randomized). Participants were told, “It’s your turn to help share these stickers with these two kids. Can you share these stickers so that each person has the same?” In each trial, the experimenter moved the computer cursor over a sticker one at a time and asked the participant which recipient the participant wanted to give the sticker to. This continued until all stickers were shared. If participants did not share all the stickers, they were reminded that they needed to share every sticker and specifically asked what they wanted to do with the remaining stickers. Experimenters administering the test trials were not blind to condition, as the same experimenter conducted both the worked example phase and the subsequent test trials. However, experimenters followed a standardized script throughout the study. In a small number of trials, participants indicated that they were finished sharing despite extra stickers remaining. In these cases, the experimenter moved on to the next trial. These “incomplete” trials comprised less than 1% of all trials and were excluded from analyses.

After the Similar block, participants experienced the Dissimilar block, where instructions were identical except they were asked to share stickers equally amongst three recipients—one in green, one in orange, and one in purple. Again, participants completed 3 trials of varying difficulty levels dividing 6 (easy), 9 (medium), and 15 (hard) stickers across the 3 recipients (order of trials randomized).

### 2.3. Data Analyses

If a child failed to follow instructions and distribute all of the stickers in a given trial as described above, data from that trial was excluded from analyses. This resulted in the exclusion of less than 1% of the data.

Performance in the test trials was calculated using an Equality Deviation score which reflected how far distributions deviated from an equal split. For each trial, the score was calculated as the sum of the absolute differences between the number of items each recipient received and the ideal equal split. Thus, larger deviation scores reflect more unequal (i.e., less fair) distributions, whereas smaller scores reflect more accurate (i.e., more fair) distributions. In the Similar block (two recipients), the ideal split was half of the total items. For example, in the 6-item trial (where the ideal split is 3-3), if a child produced a 4-2 split, the deviation score was (|4-3| + |2-3|) = 2. In the Dissimilar block (three recipients), the ideal split was one-third of the total items. For example, in the 6-item trial (where the ideal split was 2-2-2), if a child produced a 3-1-2 distribution, their score was (|3-2| + |1-2| + |2-2|) = 2. To ensure that extreme outliers did not disproportionately influence this calculation, maximum deviation scores were capped at the value of the ideal equal split (e.g., a 15-0-0 split in the 15-item Dissimilar trial would be given a score of 5). Because the possible range of deviation scores necessarily differed across set sizes (e.g., the maximum possible deviation score was determined by the size of initial set), analyses were conducted separately for each set size. Age was treated as a categorical variable (4-, 5-, and 6-year-olds) rather than continuously to examine potential non-linear developmental differences across age groups.

Participant responses to the open-ended follow-up questions asked during the worked example manipulation were transcribed and coded for all three conditions (Correct, Incorrect, and Baseline). Specifically, their responses were coded for whether participants described (1) the *process* of the division or (2) the outcome or anything else that did not fit into the process category. Qualitative coding was done by two research assistants, and a percent agreement reliability score was calculated. Percent agreement scores were above 73.55% for all trials. All disagreements were resolved by a third independent coder.

## 3. Results

### 3.1. Similar Block

#### 3.1.1. Proportion of Equal Dividers

First, we examined the proportion of children who distributed resources evenly across in the Similar block. Across all set sizes, a majority of children in each age group produced equal distributions (see Figure S1 in the Supplemental Materials). Separate Chi-square tests were run within each set size, revealing no significant differences in the proportion of children who produced even distributions across the three conditions for each of the three set sizes (all *p*s > 0.18).

#### 3.1.2. Equality Deviation Scores

To examine whether children’s performance differed as a function of Condition (Correct, Incorrect, or Baseline) and Age (in years: 4, 5, or 6 years), we conducted between-subjects ANOVAs on Equality Deviation scores for each set size (small, medium, and large) separately.^3^

In the small-set-size trials, there was a marginal Condition x Age interaction (*F*(4, 191) = 2.10, *p* = .083, η^2^_p_ = 0.04), revealing that 5-year-olds performed more accurately in the Incorrect condition (*M* = 0.10, SE = 0.17) compared to the Baseline condition (*M* = 0.58, SE = 0.15; *p* = .031), with no other condition differences in the other age groups.

Analyses of Equality Deviation scores from the medium-set-size trials did not reveal any significant main effects or interactions (*p*s > .214), indicating that children’s sharing did not differ across conditions or ages.

**Figure 3:**
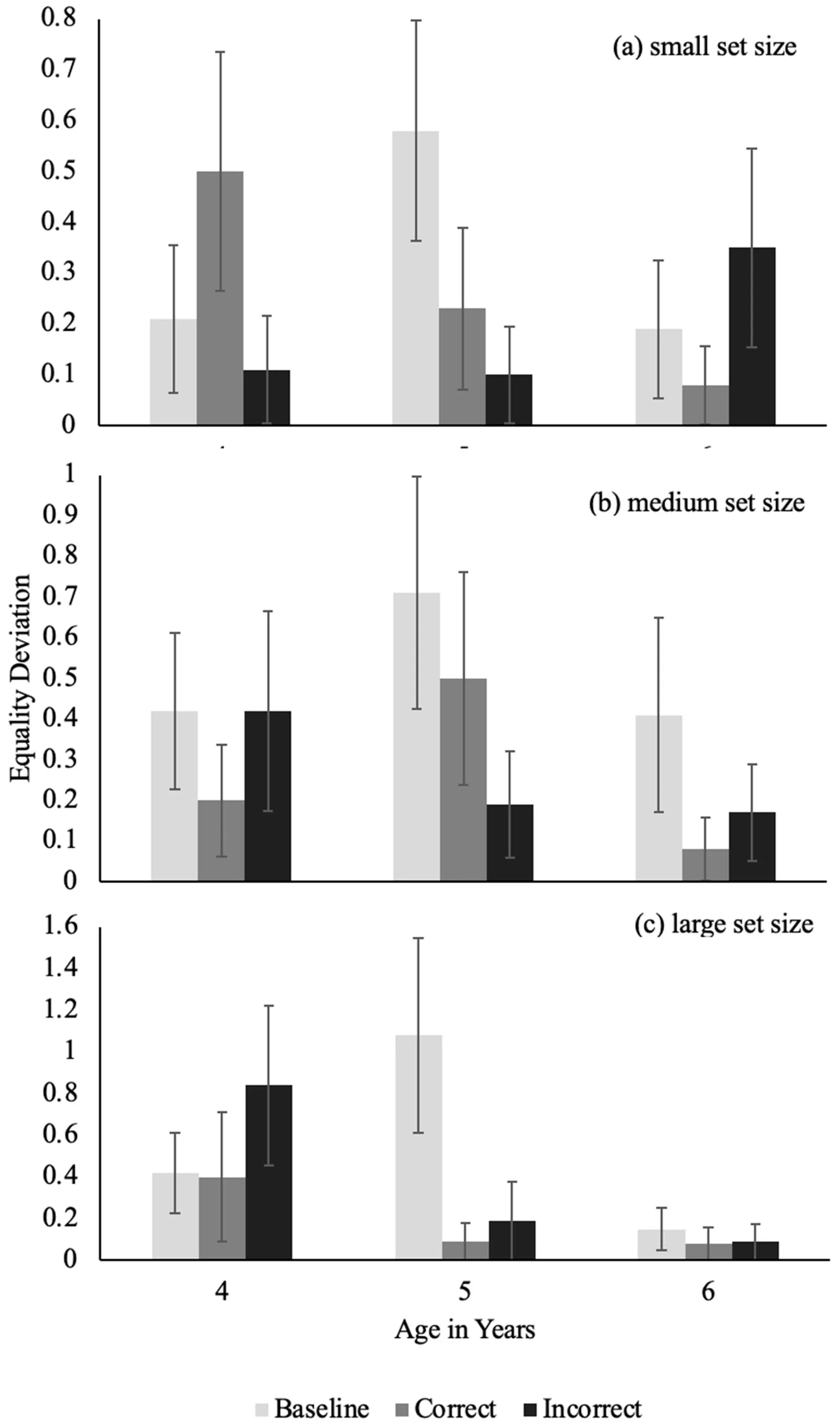
*Mean Equality Deviation scores in each set size in the Similar sharing block. Note*: Mean Equality Deviation scores by Age and Condition for each set size in the Similar block. Lower scores indicate more accurate (equal) divisions. Panels depict (**a**) small set size (maximum deviation score of 3), (**b**) medium set size (max score of 5), and (**c**) large set size (maximum score of 6). Error bars represent standard error.

Analyses of the Equality Deviation scores for the large-set-size trials revealed a marginal main effect of Age (*F*(2, 192) = 2.92, *p* = .056, η^2^_p_ = 0.03) and a marginal Condition x Age interaction (*F*(4, 192) = 2.19, *p* = .072, η^2^_p_ = 0.04), revealing that the 5-year-olds performed better in the Correct (*M* = 0.09, SE = 0.25) and Incorrect conditions (*M* = 0.19, SE = 0.25) compared to the Baseline condition (*M* = 1.08, SE = 0.24; both *p*s < .05) with no difference between the Correct and Incorrect conditions, (*p* > .77). There were no differences between conditions for the 4- and 6-year-olds (*p*s > .23). There main effect of Condition did not reach significance (*p* > .190; Figure 3).

### 3.2. Dissimilar Block

#### 3.2.1. Proportion of Equal Dividers

We again examined the proportion of children who equally divided the resources across age groups and conditions in the Dissimilar block. Across all set sizes, a majority of children produced equal distributions (see Figure S2 in the Supplemental Materials). Separate Chi-square tests were run within each set size, revealing no significant differences for the small or medium set sizes (*p*s = 0.132 and 0.67, respectively). For the large set size, there was a marginal effect of Condition, χ^2^(2) = 5.52, *p* = 0.063, with children in the Correct (82.1%) and Incorrect (85.7) conditions tending to distribute equally more often than the children in the Baseline condition (70%).

#### 3.2.2. Equality Deviation Scores

As in the analyses for the Similar block, three parallel ANOVAs were run to examine whether performance differed as a function of Condition and Age on Equality Deviation scores for each set size in the Dissimilar block.

In the small-set-size trial, there was a marginal main effect of Age (*F*(2, 191) = 2.68, *p* = .071, η^2^_p_ = 0.03), revealing that older children were more accurate in their division abilities than younger children (4-year-olds: *M* = 0.38, SE = 0.08; 5-year-olds: *M* = 0.18, SE = 0.08; 6-year-olds: *M* = 0.14, SE = 0.07). There were no other significant effects (*p*s > 0.146).

In the medium-set-size trial, there was a significant main effect of Age (*F*(2, 192) = 4.51, *p* = .012, η^2^_p_ = 0.05), again revealing that children became more accurate in their division abilities as they aged (4-year-olds: *M* = 0.62, SE = 0.11; 5-year-olds: *M* = 0.34, SE = 0.11; 6-year-olds: *M* = 0.17, SE = 0.10). There were no other significant effects for the medium-set-size data (*p*s > .41).

**Figure 4:**
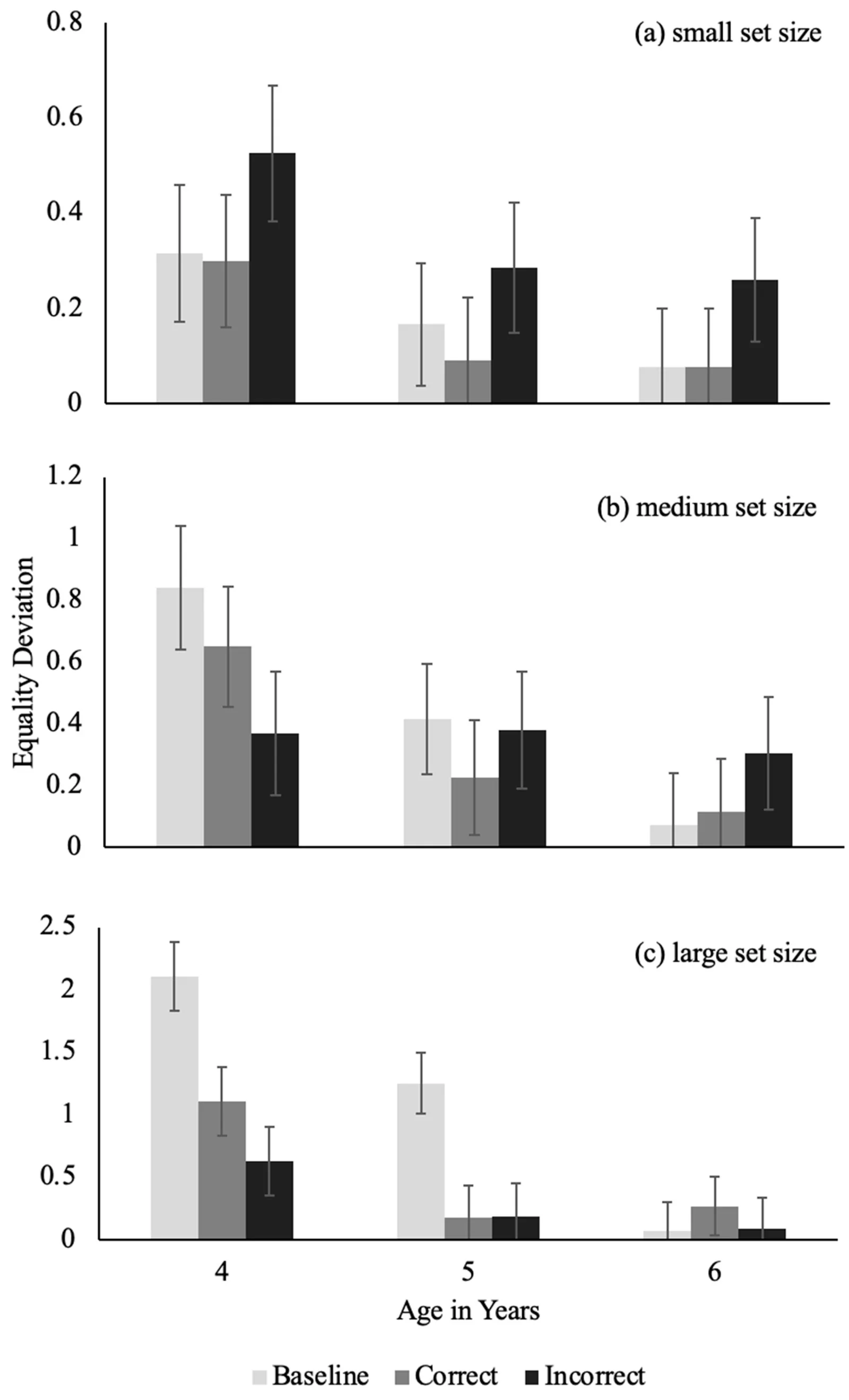
*Mean Equality Deviation scores in the small, medium, and large Trials in the Dissimilar block. Note*: Mean Equality Deviation scores as a function of Age and Condition for each set size in the Dissimilar block. Lower scores indicate more accurate divisions. Panels depict (**a**) small set size (maximum possible deviation score of 2), (**b**) medium set size (maximum possible score 3), and (**c**) large set size (maximum possible score of 5). Error bars represent standard error. Asterisks represent a significant Age x Condition interaction.

Analyses for the large set size once again revealed a main effect of Age (*F*(2, 191) = 15.01, *p* < .001, η^2^_p_ = 0.14), such that the older children in our sample were more accurate in their division abilities than younger children (4-year-olds: *M* = 1.28, SE = 0.16; 5-year-olds: *M* = 0.54, SE = 0.15; 6-year-olds: *M* = 0.14, SE = 0.14). Notably, there was also a main effect of Condition (*F*(2, 191) = 8.95, *p* < .001, η^2^_p_ = 0.09), such that performance in both the Correct (*M* = 0.52, SE = 0.15) and Incorrect (*M* = 0.30, SE = 0.15) conditions was significantly more accurate than performance in the Baseline condition (*M* = 1.14, SE = 0.14; *p*s < .05). There was no significant difference between performance in the Correct and Incorrect conditions (*p* > .30). However, both main effects were subsumed by a significant Condition x Age interaction (*F*(4, 191) = 3.20, *p* = .014, η^2^_p_ = 0.06). Follow-up analyses indicated both the 4-year-olds and the 5-year-olds performed better in the Correct (4-year-olds: *M* = 1.11, SE = 0.27; 5-year-olds: *M* = 0.18, SE = 0.25) and Incorrect conditions (4-year-olds: *M* = 0.63, SE = 0.27; 5-year-olds: *M* = 0.19, SE = 0.26) compared to the Baseline condition (4-year-olds: *M* = 2.11, SE = 0.27; 5-year-olds: *M* = 1.25, SE = 0.24; all *p*s < .05) with, again, no difference between the Correct and Incorrect conditions, (*p*s > .22). There were no differences between conditions for the 6-year-olds (*p*s > .551; Figure 4), likely due to near-ceiling performance in this age group.

### 3.3. Analyses of Children’s Explanations

#### 3.3.1. Descriptive Statistics

We first examined the general descriptive patterns in children’s explanations across all three conditions (see Table 1). Participants were given credit for a *Process* explanation if they mentioned the sharing process in at least one of the worked example videos. When asked to describe what the agent did to share correctly or incorrectly, children in both the Correct and Incorrect conditions most frequently referenced the process of sharing as opposed to the outcome of the sharing process (*p*s < .05, binomial statistics; see Table 1).

Additional analyses focused exclusively on the Correct and Incorrect conditions, examining whether participants mentioned the division process in at least one of the worked example videos (1 = mentioned process; 0 = no mention of process). For example, one child in the Correct condition explained “He grabbed one every time” reflecting a process-focused response, whereas another child in the same condition noted “He did four and four” reflecting an outcome-focused strategy. The Baseline condition was excluded from these analyses as children were simply asked to identify the colors of the agents’ shirts, a topic unrelated to division strategy^4^. We conducted additional between-subject ANOVAs to examine the effects of Age (4-, 5-, and 6-year-olds), Condition (Correct and Incorrect) and Process Explanation (present/absent) on Equality Deviation scores in each set size.

#### 3.3.2. Similar Block

In the Similar block, there were no significant main effects or interactions in the small or medium set sizes (*p*s > .096).

**Figure 5:**
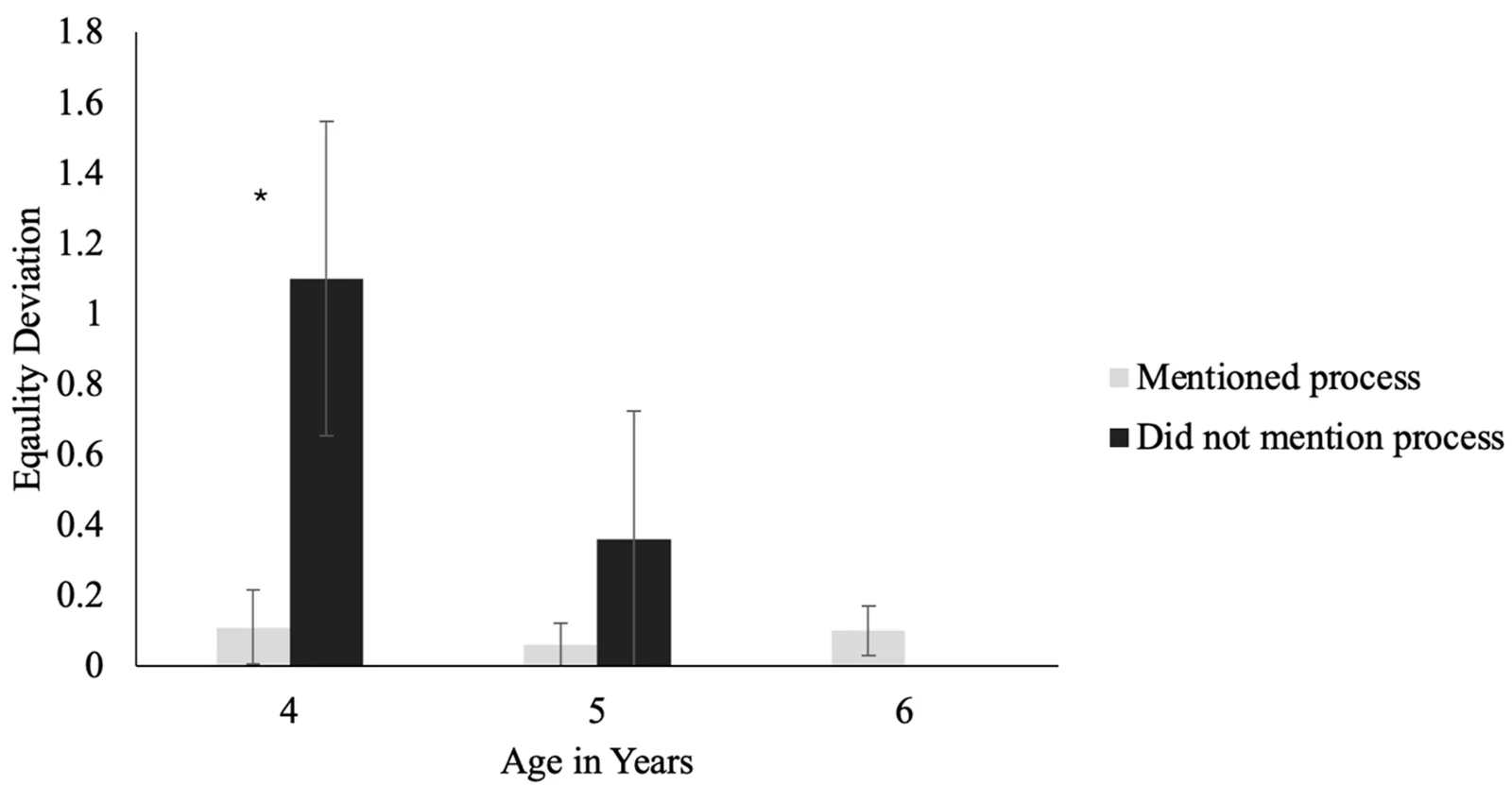
*Equality Deviation Scores as a function of set size and procedural responses in the Correct and Incorrect conditions. Note:* Mean Equality Deviation scores shown separately for children who mentioned process versus those who did not, in the largest set size group. Lower scores indicate more accurate dividing. The asterisk represents a significant Age x Process interaction. Error bars represent standard error.

Data from the large-set-size trial of the Similar block, however, revealed significant main effects of Age (*F*(2, 199) = 4.61, *p* = .012, η^2^_p_ = 0.07) and of Process Explanation (*F*(1, 119) = 7.19, *p* = .008, η^2^_p_ = 0.06). Children who referenced the division process (*M* = 0.08, SE = 0.10) performed more accurately than those who did not reference the process (*M* = 0.57, SE = 0.15), but an Age x Process interaction (*F*(2, 119) = 4.71, *p* = .011, η^2^_p_ = 0.07) revealed that this effect was driven by the youngest age group. Four-year-old children who referenced the division process (*M* = 0.08, SE = 0.21) performed more accurately than those who did not reference the process (*M* = 1.31, SE = 0.21; *p* < .001), but this was not the case for the 5- and 6-year-old children (*p*s > .28; Figure 5).

Importantly, there was also a main effect of Condition (*F*(1, 119) = 4.25, *p* = .041, η^2^_p_ = 0.03), such that children in the Correct condition (*M* = 0.14, SE = 0.13) performed more accurately than children in the Incorrect condition (*M* = 0.51, SE = 0.13). Additionally, there was a marginal Condition x Process interaction (*F*(1, 119) = 3.80, *p* = .054, η^2^_p_ = 0.03), such that, in the Incorrect condition, children who referenced the division process (*M* = 0.09, SE = 0.13) performed more accurately than those who did not reference the process (*M* = 0.93, SE = 0.22; *p* = .001). However, explaining the division process was unrelated to the accuracy in the Correct condition (mention of process: *M* = 0.07, SE = 0.15; no mention of process: *M* = 0.21, SE = 0.21; *p* = .605).

#### 3.3.3. Dissimilar Block

The same three ANOVAs were run for data from the Dissimilar blocks. There were no significant effects or interactions for data from the small and medium set sizes (*p*s > .092). In the large set size, there was a main effect of Age (*F*(2, 118) = 6.59, *p* = .002, η^2^_p_ = 0.10), revealing that children became more accurate in their division abilities as they aged (4-year-olds: *M* = 0.85, SE = 0.16; 5-year-olds: *M* = 0.18, SE = 0.16; 6-year-olds: *M* = 0.11, SE = 0.17). No other main effects or interactions reached significance (*p*s > .165).

## 4. Discussion

In this study, we examined whether children’s division performance was influenced by first explaining correct or incorrect worked examples and whether these effects varied as a function of age and task difficulty. Overall, we found that worked examples were effective under certain conditions, providing clear support for H1. That is, children in both the Correct and Incorrect worked example conditions showed improved division performance relative to children in the Baseline condition. In particular, this effect was noted when the task placed higher cognitive demands on children’s emerging division abilities. Whereas condition effects were minimal in the Similar block, where most children performed at high levels across all the set sizes, condition effects emerged in the Dissimilar block, particularly for younger children and for the largest set size, presumably where children were most challenged by the task. Thus, consistent with H3, we found worked example benefits emerged primarily on the most challenging trials and among the youngest children who had yet to master division. Together, these findings suggest that worked examples and explanation prompts may be most beneficial when children are reaching the limits of their current competence.

First, it is important to note that the 6-year-old children in our study performed at or near ceiling across all conditions and set sizes. This pattern is consistent with prior work showing that by this age children have typically mastered the verbal counting procedure and can reliably implement fair sharing strategies (Chernyak et al., 2016; Jacobs et al., 2022). Given their numerical competence, the additional support in the form of worked examples offered limited benefits (Mix & Cheng, 2012).

Among the four- and five-year-olds, however, worked examples appeared to support children’s performance. This was most apparent in the Dissimilar block, where children had to divide resources among three recipients. The added cognitive demands of tracking an extra recipient and coordinating turn-taking in this novel format likely made this condition more difficult, allowing for the benefit of worked examples to emerge. Four- and five-year-old children in both the Correct and Incorrect conditions produced lower Equality Deviation scores in the more difficult large-set trials compared to the Baseline condition. These results are consistent with prior work suggesting that worked examples are most effective when learners have a sufficient base understanding but have not yet fully mastered the skill (Atkinson et al., 2000; Booth et al., 2013).

Notably, our findings revealed improvements for children in the Correct and Incorrect condition alike: the incorrect worked examples appeared just as effective as the correct examples. Moreover, we did not find the effect of correct and incorrect manipulations to differ as a function of age. Thus, our results were not consistent with H2, which predicted a differential pattern with development. However, this pattern is consistent with work demonstrating that evaluating mathematical *procedures* such as explaining why a solution is incorrect, can promote conceptual understanding by drawing attention to underlying strategies rather than more surface-level outcomes (Siegler, 2002; Große & Renkl, 2007). Although a meta-analysis found that correct worked examples may be more effective than incorrect worked examples (Barbieri et al., 2023), there are possible explanations for why we did not find this to be the case. Given that no studies to date have explored the effects of worked examples with children this young, it is possible that a general focus on *process* confers benefits for younger children’s conceptual learning of division. Also, to note, we operationally defined an incorrect worked example differently than in past work, such that the procedural error in that video was ultimately corrected, resulting in an equal outcome. Therefore, it is possible that only a subset of children registered the procedural deviation at all. Consistent with this, we found that children who spontaneously referenced the division process performed more accurately than those who did not, but only in the Incorrect condition (Section 3.3.2). This suggests that it was specifically the children who noticed and could articulate the error who benefited, rather than the incorrect example benefiting all children uniformly. Both correct and incorrect worked examples were effective for children this age, suggesting that highlighting procedural strategies may be enough to support early division learning. Future research with more trials and across a wider age range is needed to examine whether effects of correct versus incorrect examples emerge as children’s division knowledge matures.

The results of our analyses of children’s explanations suggest that the usefulness of worked examples may lie in drawing attention to the math procedures themselves. Aligning with our fourth hypothesis (H4), when asked why the agent shared correctly or incorrectly, children who referenced the process of sharing rather than the outcome or some other factor performed more accurately. Notably, this was only the case in the large set size for the youngest participants (four-year-olds), where children who spontaneously described the process of division outperformed those who did not mention the process. Though this was not found in the older age groups, this could be due to a ceiling effect in performance, particularly for the six-year-olds. These findings suggest that our worked examples may have succeeded by highlighting *how* the sharing was done, not just *what* the outcome was, serving as a powerful mechanism for improving math reasoning in early childhood. This is consistent with prior work that emphasizes the importance of process-focused instruction in early education (Fyfe et al., 2014; Rittle-Johnson, 2006; Rittle-Johnson et al., 2017). Together, these findings suggest that worked examples may be most effective not just because they reduce cognitive load but because they direct children’s attention toward task-relevant procedural structure during moments in which their own strategies are insufficient.

From a Cognitive Load Theory perspective (Sweller, 2011), worked examples help reduce working memory demands by breaking down a solution into more manageable steps, allowing learners to focus on each component (Renkl, 2014). This framework also helps explain our finding that children who spontaneously referenced the division process performed more accurately than those who did not. By articulating the procedural steps, this may have reduced the cognitive demands of the task. In the present study, worked examples appeared most beneficial when task demands were high, as in the large Dissimilar trials for the younger children. More specifically, the worked example may have allowed children to focus on tracking the procedure, how items were distributed across recipients, rather than also tracking the number of items shared. This may explain why younger children benefited primarily in the most difficult trials, whereas the oldest children (6-year-olds) who were already performing near ceiling saw no improvements. When cognitive demands were relatively low, worked examples offered little to no added benefit, suggesting that children relied on their existing intuitive division strategies until their cognitive resources are exceeded. Additionally, we suspect that children need some minimum level of prior knowledge (in this case, an understanding of small set counting) to meaningfully benefit from a worked example. If a child does not have this underlying understanding, a worked example may be too abstract to scaffold their learning.

Taken together, these findings contribute to the literature on worked examples by showing their potential in early math learning, when children are at an age where they are still developing foundational numerical concepts. Most prior research on worked examples and self-explanation has focused on older, school-aged children learning more abstract mathematical procedures such as algebra or problem solving (Renkl, 2014; Booth et al., 2013). In contrast, the present study examined preschool-aged children learning the core foundational process of division embedded within a socially meaningful sharing context and found that even young children can benefit from these structured worked examples when the task demands are close to their level of understanding.

Given the age range of our sample, we modified our worked examples to be short, engaging videos for the children. To ensure that the children were actively processing the information, we asked them questions about the videos, prompting them to reflect on the sharing procedures they observed. This is a form of self-explanation that has been shown in prior research to be effective in promoting deeper encoding and transfer of mathematical knowledge (Booth et al., 2013; McNeil & Fyfe, 2012). Our findings suggest that our manipulation was effective and that such benefits may emerge earlier in development than previously documented.

However, it should be noted that both of our Correct and Incorrect conditions required the children to engage in self-explanation—that is, they were asked to explain the procedures in both cases. As such, it could be argued that we simply compared the manipulation of asking the children to engage in self-explanation in either the Correct or Incorrect condition to a Baseline condition in which they observed the same worked examples but were not required to analyze the procedure. This does limit our ability to examine the differences between correct and incorrect examples. Nevertheless, it is notable that this minimal manipulation was sufficient to influence participants subsequent performance, such that we found evidence that discussing the division procedure—whether correct or incorrect—had an impact on their subsequent division performance. Based on prior work revealing that children of this age are highly sensitive to adult models in numerical tasks (Posid & Cordes, 2018), it is likely that simply viewing these videos led to boosted performance for all participants in our tasks. Additional studies may want to explore how children would have performed in our tasks without viewing the videos beforehand to see how much these videos may have impacted performance and to more precisely assess the contribution of observation versus prompted explanation.

We also did not code children’s gestures during their explanations, focusing instead on their verbal responses. Given that gesture could possibly convey procedural understanding, future work should code gesture alongside verbal explanation to more fully capture how young children understand the sharing process.

These findings also have some clear implications for early educational practices. The worked example approach used in this study is simple and easily adaptable for use in classroom settings, such that teachers can demonstrate a division or counting strategy and then prompt children to explain what was done correctly or incorrectly. We chose a sharing context because prior research indicates that sharing provides a natural and effective entry point into children’s understanding of division, as they require the coordination of quantity, equality, and procedure (Hamamouche et al., 2020; Squire & Bryant, 2002b). Importantly, highlighting procedures through worked examples and explanation prompts is not specific to sharing contexts and is likely applicable to a broad range of early mathematical concepts. It is an open question whether worked examples generalize similarly across early math concepts that vary in cognitive demand versus those that are primarily skills-based. For example, sharing among a varying number of recipients places different cognitive demands on a child compared to a counting task that is more skill-based. Whether worked examples confer the same benefits across both of these types of tasks has yet to be answered.

Overall, our findings demonstrate that correct and incorrect worked examples can help scaffold young children’s learning of simple division. Exploratory analyses suggest that improvements, at least in the Incorrect condition, were likely driven by our manipulation highlighting the *process* of dividing equally, rather than drawing attention to the outcome of the division. By directing children’s attention to how equal distributions are created, worked examples can help support the development of foundational preschool mathematical understanding, which is critical for later learning.

## Figures and Tables

**Table 1:** *Percentages of children’s explanation types by Condition*.

Condition	Process	Not Process
Correct	66.2%	33.8%
Incorrect	73%	27%

*Note: Process* responses referenced how the agents distributed the items (e.g., counting or alternating), whereas *Not Process* responses included attempts to answer the question that did not reference either the sharing process or the outcome.

## Data Availability

Study materials including video stimuli are publicly available on the Open Science Framework (OSF) at https://osf.io/vsbgh/overview?view_only=54554e96dfac4788adf6d51a8069ed79 (accessed on 6 April 2026). Data will be made available upon reasonable request to the corresponding author.

## Notes

1 All children participated in English, except one child who participated in Portuguese with a fluent Portuguese-speaking researcher using the same study protocol and scripts (translated to Portuguese). All children were enrolled in school, and no children were flagged as having comprehension difficulties.

2 The genders of the two agents were intentionally ambiguous such that the agents’ faces were not shown (videos cut off at the shoulders), and gender-neutral names were used. However, the agents’ voices were female.

3 To verify that the testing modality did not influence these results, we reran each ANOVA reported in Section 3.1.2 and Section 3.2.2 with modality (Zoom vs. in-person) added in as a third factor. There were no significant main effects or interactions involving modality (*p*’s > .19), and the pattern of results did not change. Therefore, modality was not included as a factor in the analyses reported in the main text. We ran analyses exploring whether experiencing the correct or incorrect video first influenced the results. There were no significant main effects or interactions involving order (*p*’s > .161).

4 In the Baseline condition, children were asked to identify the agent’s shirt color rather than evaluate the sharing behavior. Among these children (*n* = 70), 65.7% correctly identified the agent’s shirt color in the 8-item video, and 72.9% correctly identified the agent’s shirt color in the 12-item video. Importantly, correct identification of shirt color was inconsequential to their performance on the practice and test trials.

## Supplementary Materials

The following supporting information can be downloaded at: https://www.mdpi.com/article/10.3390/jintelligence14090192/s1, Figure S1: Proportion of Children Producing Equal Distributions in the Similar Block; Figure S2: Proportion of Children Producing Equaling Distributions in the Dissimilar Block.

## Abbreviations

The following abbreviations are used in this manuscript:

ANOVA	Analysis of Variance
CLT	Cognitive Load Theory
SE	Standard Error
ZPD	Zone of Proximal Development
