# Supplementary material for: The Role of Worked Examples in Supporting Early Math Learning
Source: J Intell. 2026 Sep 1;14(9):192. doi: 10.3390/jintelligence14090192 (PMC13608103; doi:10.3390/jintelligence14090192)
Supplement: Supplementary file 1 [file jintelligence-14-00192-s001.zip › jintelligence-4400798-supplementary.pdf]

Supplementary Material

Figure S1

Proportion of Children Producing Equal Distributions in the Similar Block

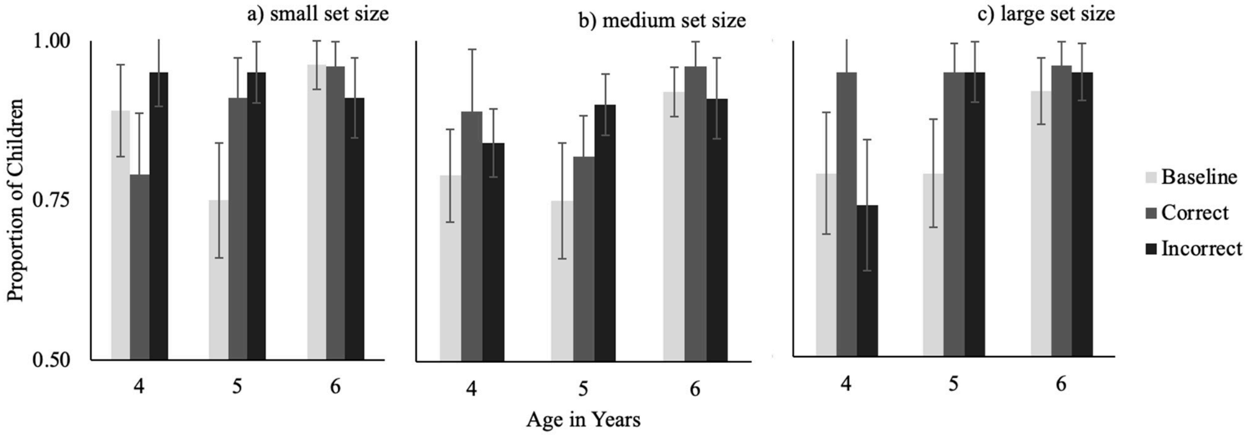

Note. The values reflect the proportion of children within each set size group who divided the stickers evenly between two recipients on each trial. Panels depict (a) small set size (b) medium set size and (c) large set size.

Figure S2

Proportion of Children Producing Equaling Distributions in the Dissimilar Block

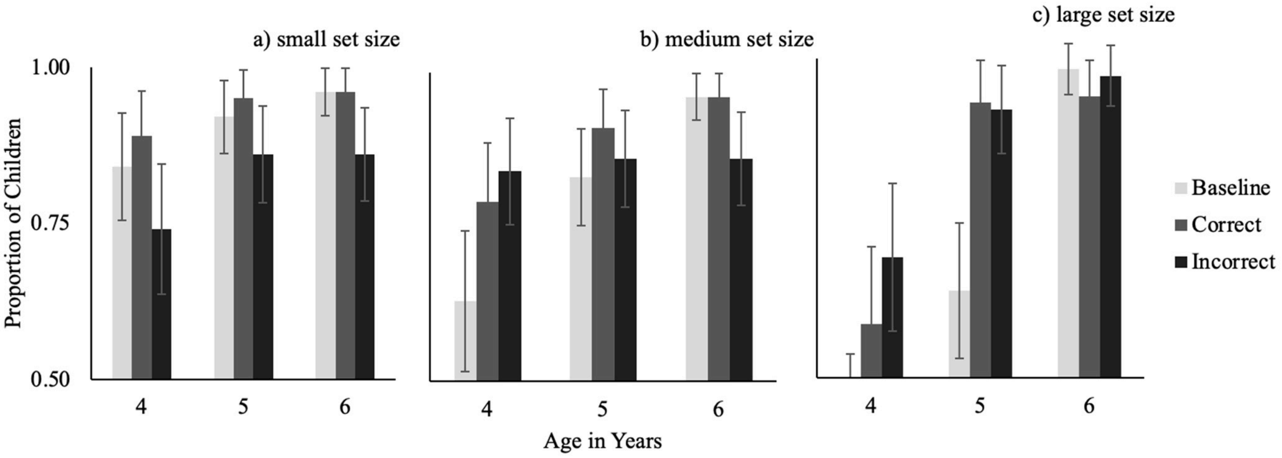

Note. The values reflect the percentage of children within each set size group who divided the stickers evenly between two recipients on each trial. Panels depict (a) small set size (b) medium set size and (c) large set size.
